# Abiotic microcompartments form when neighbouring droplets fuse: an electrochemiluminescence investigation†

**DOI:** 10.1039/d2sc06553c

**Published:** 2022-12-27

**Authors:** Silvia Voci, Thomas B. Clarke, Jeffrey E. Dick

**Affiliations:** a Department of Chemistry, Purdue University West Lafayette IN 47907 USA jdick@purdue.edu; b Elmore Family School of Electrical and Computer Engineering, Purdue University West Lafayette IN 47907 USA

## Abstract

Many studies have shown chemistry proceeds differently in small volumes compared to bulk phases. However, few studies exist elucidating spontaneous means by which small volumes can form in Nature. Such studies are critical in understanding the formation of life in microcompartments. In this study, we track in real-time the coalescence of two or more water microdroplets adsorbed on an electrified surface in a 1,2-dichloroethane continuous phase by electrogenerated chemiluminescence (ECL) imaging, uncovering the spontaneous generation of multiple emulsions inside the resulting water droplets. During the fusion of adsorbed water droplets with each other on the electrode surface, volumes of organic and water phases are entrapped in between and detected respectively as ECL not-emitting and emitting regions. The diameter of those confined environments inside the water droplets can be less than a micrometer, as described by scanning electron microscopy data. This study adds a new mechanism for the generation of micro- and nano-emulsions and provides insight into confinement techniques under abiotic conditions as well as new potential strategies in microfluidic devices.

## Abstract 2

The collision of two or more water microdroplets adsorbed on an electrified surface has been tracked by ECL imaging, uncovering the dynamic complexity of the resulting water microdroplet.

## Graphical abstract

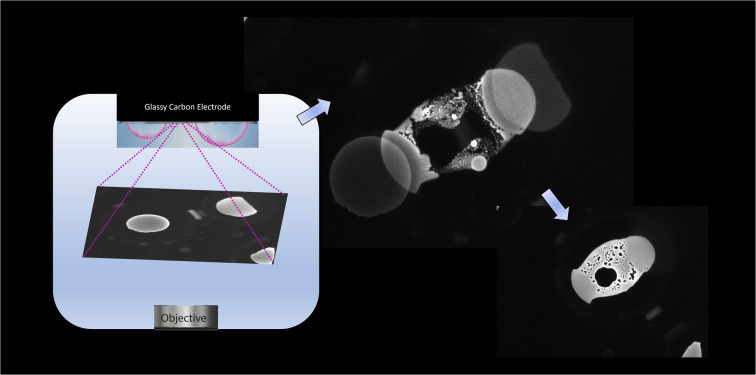

In multiphasic systems, drastic differences in reactivity have been reported over the last years compared to bulk solutions.^1^ The presence of multiple interfaces confines reactants and has exceptional consequences on the kinetics and physicochemical properties of the chemical environment within.^2,3^ Many groups have reported on unusual chemical reactivity in microliter water droplets. Cooks and co-workers^4,5^ have demonstrated that chemical reactions are accelerated in aqueous microdroplets generated by electrospray. Griffiths and colleagues^6,7^ showed the importance of reaction adsorption to a liquid|liquid interface in the acceleration of a fluorogenic reaction. Zare's team^8^ has studied spontaneous reduction and peroxide formation at the liquid|air interface, and they have investigated the role the electric field might play at this and liquid|liquid boundaries.^9,10^ In addition to the studies of the acceleration of organic and small molecules reactions, multistep biomolecular reactions confined in microdroplets have also been reported.^11,12^ In particular, Zare and coworkers observed a 7.5-milion times improvement in the speed of the digestion of antibody by a protease enzyme.^13^ The confinement of water phases plays an important role in biological systems.^14^

In nature, an increasing number of observations shows that some sub-cellular systems can behave as liquid-like droplets.^15^ Compartmentalization inside cells is indeed not restricted to the use of physical barriers such as a lipid bilayer. Liquid–liquid phase separation can occur, allowing the concentration of certain factors and an easier exchange with the rest of the environment. The study of such membrane-less organelles is of fundamental importance in the elucidation of prebiotic conditions and of the development of some human diseases.^16^ Nucleoli, subnuclear membrane-less systems composed of RNA and proteins, can behave as active liquid-like droplets and their number and shape are inevitably modified in many cancer cells. Even their coalescence is related with an increase in the number of internal compartments and substructures.^17,18^ The physical processes that give rise to the assembly of those systems can be studied by using water droplets as simplified models.^3,14^

In this work, we applied the electrogenerated chemiluminescence (ECL) imaging technique to the study of the generation of confined environments inside sessile water droplets by tracking their coalescence with time. ECL is a kind of chemiluminescence triggered by an electrochemical stimulus.^19^ Its dual nature as both a photochemical and electrochemical phenomenon makes ECL-based microscopy complementary to fluorescence and optical microscopies in the visualization of physical, biological, and chemical events.^20,21^ The first heterogenous electron transfer step in ECL limits the extension of ECL emission to the maximum diffusion and lifetime of the produced ECL reactants (a few micrometers from the electrode surface).^22^ ECL microscopy is considered a surface-confined imaging technique and affords higher resolution of events occurring at the interface between the electrode and the object under study.^23,24^ In the context of multiphasic systems, ECL has been used to investigate collisions of droplets on an electrode surface,^25,26^ to study the confinement phenomenon in oil droplets stabilized by luminophore-decorated microgels^27^ and to visualize phase boundaries in sessile water droplets.^28^ In this latter case, the presence of a three-phase boundary must be added as a descriptor together with the surface tension which favours droplets coalescence and the viscosity that slows the process.^29^

Previously, our group reported the entrapment of volumes of organic continuous phase inside water droplets adsorbed on an electrode surface by ECL microscopy.^30^ In that work, we observed an increase in the number of organic inclusions inside the water phase with bigger dimensions of the water droplets. Here, the imaging of the evolution of coalescing sessile water droplets over a few seconds time interval by ECL microscopy reveals that coalescence of smaller water droplets is responsible for the generation of the previously reported organic phase environments confined inside the resulting water droplet. Moreover, the high resolution of the ECL microscopy compared to optical microscopy highlights the presence of confined aqueous compartments inside the coalesced water droplet. This complex system contains membrane-less confined compartments of different chemical nature (hydrophilic and hydrophobic), and results from the spontaneous coalescence of water droplets adsorbed on the electrode surface. Thus, our results give rise to a new mechanism by which abiotic confinement can arise in nature on a heterogeneous interface.

The emulsion under study was composed by a continuous phase of 0.1 M tetrabutylammonium perchlorate in 1,2-dichloroethane and water droplets loaded with 10 mM tris(bipyridine)ruthenium(ii) dichloride and 50 mM sodium oxalate, which are both insoluble in the organic phase. Water droplets were allowed to stochastically adsorb to a glassy carbon electrode (radius 1.5 mm) and by applying an anodic potential of 1.5 V *vs.* Ag/AgCl (see Fig. S1† for an example of electrochemical signal), ECL emission was generated inside the aqueous phase through the following reactions (eqn (1)–(6)):^31–33^

1 [Ru(bpy)_3_]^2+^ − e^−^ → [Ru(bpy)_3_]^3+^

2 (C_2_O_4_)^2−^ − e^−^ ⇄ (C_2_O_4_)˙^−^

3 (C_2_O_4_)^2−^ + [Ru(bpy)_3_]^3+^ → (C_2_O_4_)˙^−^ + [Ru(bpy)_3_]^2+^

4 (C_2_O_4_)˙^−^ → CO_2_˙^−^ + CO_2_

5 [Ru(bpy)_3_]^3+^ + CO_2_˙^−^ → [Ru(bpy)_3_]^2+^* + CO_2_

6 [Ru(bpy)_3_]^2+^* → [Ru(bpy)_3_]^2+^ + *hν*

The ECL emission was then collected by an imaging system, as represented in Fig. 1.

**Fig. 1:**
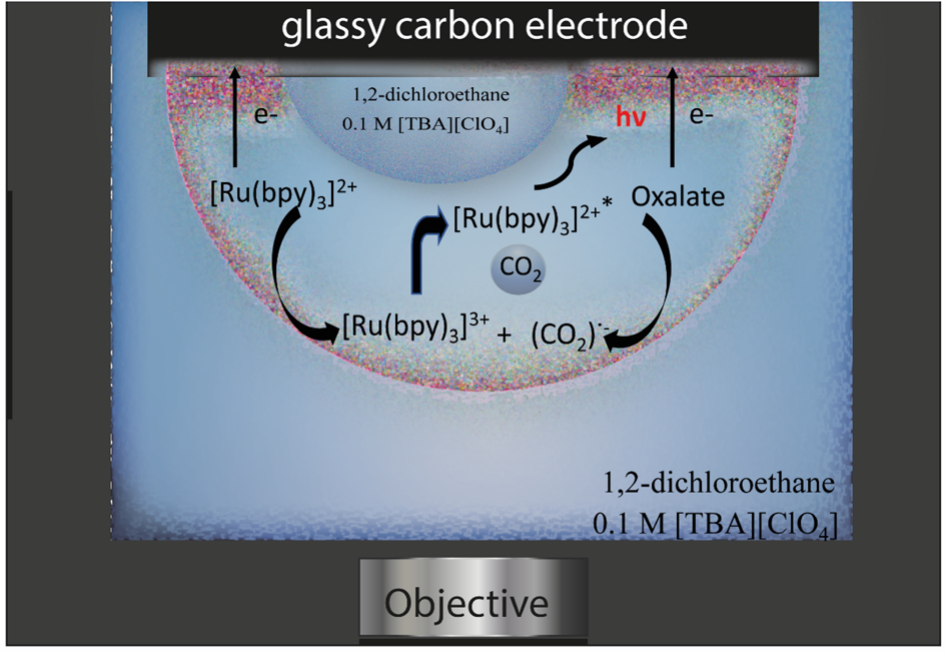
Schematic representation of the ECL imaging experiment and of the ECL mechanism.

The ECL emission generated from the aqueous phase|electrode interface was detected in reflection mode by an epifluorescence microscope system. As already described in a previous publication,^30^ regions inside the water droplets occupied by pockets of 1,2-dichloroethane were observed and, when in contact with the electrode, did not emit ECL, since both the ECL luminophore and the coreactant were not present in the organic phase. The generation of confined environments inside water droplets was tracked by sequentially recording ECL images on a glassy carbon working electrode in the presence of adsorbed water droplets. Even though large overpotentials will influence the surface of glassy carbon, the effect is minimized compared to platinum and gold surfaces.^34^ On platinum and gold, the ECL signal is convoluted with surface oxidation and water oxidation. Therefore, we chose glassy carbon as the material to pursue this study. Microcompartments are also present on a platinum electrode, but the lower ECL intensity and the shorter time extension of the emission (as reported in Fig. S2†) do not permit to follow the generation of those microcompartments starting from smaller droplets fusion. Fig. 2 shows how the coalescence of two water droplets encapsulates different confined regions composed of DCE continuous phase (dark areas) and water phase (a schematic representation of the ECL imaging set-up is reported in Fig. S3c†). Previous studies reveal that coalescence of sessile droplets does not follow a universal behaviour. The rate of the phenomenon depends on the size of the droplets, the wettability of the surface, the nature of the continuous phase, and other factors. Coalescence dynamics is governed by Navier–Stokes equations, which allow one to model this fluid mechanics phenomenon upon defining appropriate boundary and initial conditions.^35^ Different energetic contributions must be considered during the process, such as average curvature of the droplet, surface energy release, kinetic and gravitational energies, viscous dissipation, and contact line dissipation.^36^ Those energetic components sharply change during the coalescence event. The first step in coalescence is the generation of the liquid bridge between the two droplets, which expands, usually in milliseconds, because of the high capillary pressure. The high-pressure difference at the interfaces between each droplet and the continuous phase drives the expansion of the bridge and the relaxation of the resulting droplet in an elliptical shape by fluid motion.^36^ It has been found that bridge expansion and relaxation proceeds linearly with time (*t*) in the direction perpendicular to the surface, while it is proportional to *t*^½^ in the direction parallel to the substrate, following the principle of mass conservation.^37^ An important dimensionless descriptor of the droplet mechanics in a biphasic liquid|liquid environment is the Weber number, which is linearly proportional to droplet dimensions, density, and inversely proportional to surface tension.^38^ When the Weber number is small, permanent coalescence events occur while higher Weber numbers lead to reflexive separation. This condition and the adsorption of several droplets on the electrode surface permitted the observation of coalescence processes in our experimental conditions. During our ECL investigation, we distinguished three major steps that have been reported previously as separate steps in both experimental results and theoretical models.^39,40^ After an initial state in which the contact line between the droplets remains static (Fig. 2a), see Time_Laps_of_Figure_2 in ESI† for the complete time laps of the coalescence event), a liquid bridge is produced by capillarity between the droplets (Fig. 2c). An intermediate is then generated, in which the liquid bridge relaxes, and the new droplet assumes an elliptical shape (Fig. 2e, g and i). Finally, the new droplet relaxed in a circular shape. The final circular droplet is visible in the bright field image in Fig. S3b†. However, the interface between the electrode and droplet still shows an elliptical shape in Fig. 2k.

**Fig. 2:**
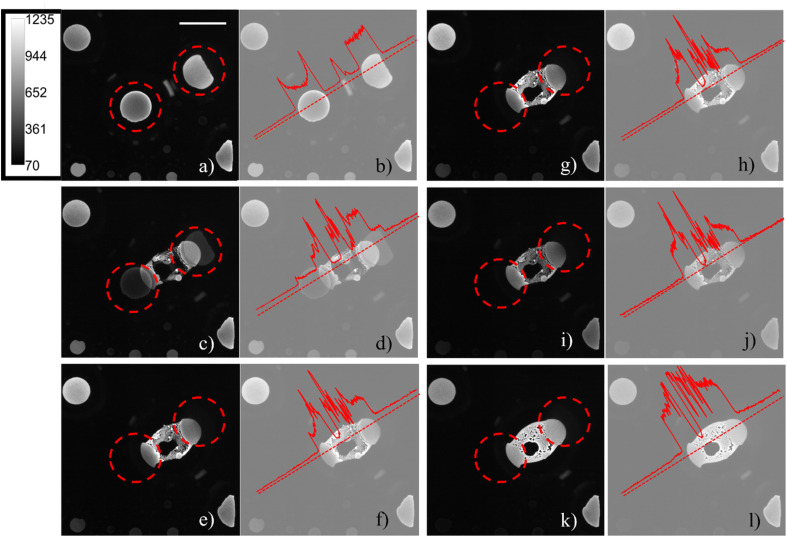
(a), (c), (e), (g), (i) and (k) ECL images of two water droplets loaded with 10 mM tris(bipyridine)ruthenium(ii) dichloride and 50 mM sodium oxalate and adsorbed on the surface of a glassy carbon electrode (*d* = 3 mm). ECL images were obtained by applying 1.5 V *vs.* Ag/AgCl on the glassy carbon working electrode and were sequentially recorded one after another on the same region of the electrode with an exposure time of 10 seconds for each image, in bottom view mode (that is the focal plane z perpendicular to the electrode surface). The intensity calibration bar is valid for all images. The dashed red circles evidence the starting position of the two droplets before coalescing. (b), (d), (f), (h), (j) and (l) Normalized intensity profiles corresponding to the ECL image on the left of each one. The dotted red straight line corresponds to the area selected for the intensity profile. Scale bar: 500 μm.

During the generation of a bridge between the initial droplets, the water phase incorporates volumes of continuous phase as well as smaller water droplets lying in between (Fig. 2c), generating confined chambers of both phases (*i.e.*, a multiple emulsion) inside the resulting water droplet. The water volumes inside the coalesced water droplet can be distinguished due to the permanence of their interface. In addition, the footprints of the water droplets which are coalescing are still visible during all the steps of the coalescence. The ECL intensity profiles (Fig. 2b, d, h, j and l), reported on the right of each ECL image, show a local maximum peak in correspondence of each distinguishable entrapped water droplet, while a decrease in ECL is visible for each detectable interface.

By observing the bright-field optical images obtained before and after the application of the potential, Fig. S3,† the passage from two water droplets to a bigger single water droplet is clearly visible, however the interface between the electrode and the water droplet is not resolved enough to distinguish the footprints of the original two water droplets. The reason for the difference between ECL and *other* optical microscopies must be found in the surface-confinement characteristic of the ECL emission. The ECL mechanism confines the emission at a maximum of few micrometres from the electrode surface, depending on the species involved. The absence of out-of-focus and scattered photons (as the ones collected in a fluorescence imaging experiment) guarantees a very high signal to noise ratio in ECL microscopy and permits to observe in details events occurring at the electrode water phase interface.^41^ A similar result can be obtained in confocal microscopy, however the use of lasers for photoexcitation may cause photobleaching and rapid events (in orders of milliseconds) can hardly be followed by confocal microscopy. In Fig. 3, the whole electrode interface is covered by a water droplet, resulting from the coalescence of all the droplets adsorbed on the electrode initially, without previous application of any potential.

**Fig. 3:**
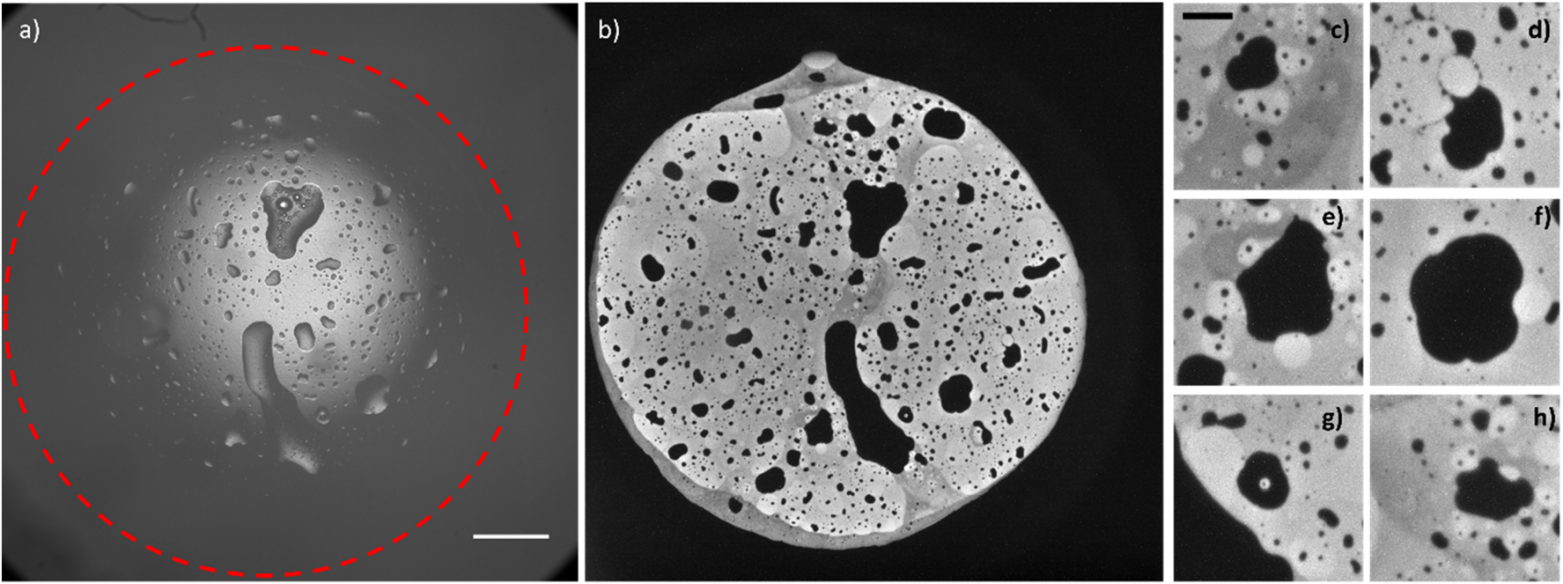
Bright-field image (a, scale bar: 500 μm) and corresponding ECL image (b) of a water droplet loaded with 10 mM tris(bipyridine)ruthenium(ii) dichloride and 50 mM sodium oxalate and adsorbed on the surface of a glassy carbon electrode (*d* = 3 mm); (c)–(h) details (scale bar: 100 μm) of the ECL image (b). ECL image was obtained by applying 1.5 V *vs.* Ag/AgCl on the glassy carbon working electrode.

The ECL image shows that the macroscopic water droplet is composed of different confined areas of both organic and water phases. A multiple emulsion is spontaneously generated on the electrode surface. Different details (Fig. 3c–h) even show areas of water in oil in water-in-oil emulsions. Although it has been demonstrated in previous publications^42,43^ that the application of an electric field influences the coalescence of droplets on a conductive surface (electrocoalescence), it seems that the low potential values applied in the ECL imaging do not drastically influence the spontaneous generation of multiple emulsions, still visible in Fig. 3 without the prior application of any potential. As mentioned above, the speed of coalescence depends on the size of the droplets. Therefore, smaller droplets will coalesce in a shorter time range. Fig. S4 and S5† show a series of sequential ECL images of smaller droplets whose coalescence was completed in about 1 second (the intensity of the ECL emission and the properties of the optical system permitted a minimum exposure time of 500 ms). We did not observe coalescence events in the oil droplets inside the water droplets. However, bigger oil droplets present defined curvatures as a “footprint” of an ensemble of smaller droplets (for example, the millimetric oil droplets reported in Fig. 3 and the oil droplet that is forming between the two droplets in Fig. S4b† and that is visible in Fig. S4c).† Therefore, we may surmise that bigger oil droplets derive from coalescence of smaller oil droplets.

Even if ECL microscopy allows a high-resolved description of the events occurring at the electrode|water droplet interface, without specific data processing^44^ the resolution cannot go beyond the diffraction limit. Specifically, in our experimental conditions we were able to detect inclusions of a minimum of about 1 μm.

We were interested in how small the inclusions could be, as the dimensions of the confined chambers largely influence the variation of the chemical reactivity. To achieve sub-micrometer resolution, we electrodeposited a metal on the electrode surface using water microdroplets that contained metal salt precursor and became adsorbed onto the electrode. Then, by using scanning electron microscopy (SEM), we were able to observe similar oil-in-water inclusions. In this separate experiment, water droplets containing 10 mM CoCl_2_ and 1 M KCl were emulsified *via* sonication into the same continuous phase used for the ECL experiments (0.1 M tetrabutylammonium perchlorate in 1,2-dichloroethane) and were allowed to collide with a glassy carbon electrode. Cobalt deposition with the water droplets was achieved *via* chronopotentiometry, wherein 50 μA of cathodic current were passed across the glassy carbon electrode for 10 s. SEM permitted the localization of the deposited cobalt. Confined areas without deposition were detected within elliptically shaped deposits (Fig. S6†), due to encapsulated regions of 1,2-dichloroethane. Several of these inclusions measured smaller than a micrometer, providing increased spatial resolution of these spontaneously formed organic entrapments. However, despite this increased spatial resolution, using SEM to visualize metal electrodeposition does not provide any ability to observe the formation of organic phase inclusions in real time. In addition, while in ECL images it is possible to distinguish water phase inclusions inside the water droplet resulting from the coalescence, SEM; instead, it only provides a fossil record of where aqueous droplets collided and coalesced on the electrode surface.

This work represents the first demonstration of spontaneous multiple emulsions generation as detected by ECL imaging. Not only does this work represent a new means by which emulsions can form, it represents a way that confined environments are built on surfaces in the environment. By using ECL as a surface-confined technique, the dynamics of sessile droplet coalescence could be resolved at the level of the interface electrode|water droplet, revealing the entrapment of water droplets and organic continuous phase areas inside the resulting droplet. The presence of multiphase confined environments inside a water droplet can represent a simplified model to investigate the biophysics and the chemical reactivity of membrane-less subcellular compartments of abiotic importance and a potential strategy in microfluidic devices development.

## Data availability

The datasets generated during and/or analysed during the current study are available from the authors on reasonable request.

## Author contributions

Silvia Voci and Jeffrey E. Dick conceived the research. Silvia Voci performed ECL imaging experiments. Thomas B. Clarke performed the SEM experiment. Silvia Voci drafted the manuscript. Jeffrey E. Dick and Thomas B. Clarke edited the manuscript. Jeffrey E. Dick revised the manuscript. Jeffrey E. Dick managed all aspects of the research.

## Conflicts of interest

There are no conflicts to declare.

## Supplementary Material

SC-014-D2SC06553C-s001

SC-014-D2SC06553C-s002
