# Correction: Abiotic microcompartments form when neighbouring droplets fuse: an electrochemiluminescence investigation

**DOI:** 10.1039/d3sc90104a

**Published:** 2023-06-29

**Authors:** Silvia Voci, Thomas B. Clarke, Jeffrey E. Dick

**Affiliations:** a Department of Chemistry, Purdue University West Lafayette IN 47907 USA jdick@purdue.edu; b Elmore Family School of Electrical and Computer Engineering, Purdue University West Lafayette IN 47907 USA

## Abstract

Correction for ‘Abiotic microcompartments form when neighbouring droplets fuse: an electrochemiluminescence investigation’ by Silvia Voci *et al.*, *Chem. Sci.*, 2023, **14**, 2336–2341, https://doi.org/10.1039/D2SC06553C.

The authors regret that their source of funding was not acknowledged in their paper. The authors acknowledge funding for this work from the National Institute of General Medical Sciences under Grant R35-GM138133.

The Royal Society of Chemistry apologises for these errors and any consequent inconvenience to authors and readers.

## Supplementary Material

