# Supplementary material for: Abiotic microcompartments form when neighbouring droplets fuse: an electrochemiluminescence investigation
Source: Chem Sci. 2022 Dec 27;14(9):2336–41. doi: 10.1039/d2sc06553c (PMC9977408; doi:10.1039/d2sc06553c)
Supplement: SC-014-D2SC06553C-s001 [file SC-014-D2SC06553C-s001.pdf]

Electronic Supplementary Material (ESI) for Chemical Science.
This journal is © The Royal Society of Chemistry 2023

Supporting Information:

Abiotic Microcompartments Form when Neighbouring Droplets Fuse: An Electrochemiluminescence Investigation

Silvia Voci[a], Thomas B. Clarke[a], Jeffrey E. Dick[a,b,*]

[a]Department of Chemistry, Purdue University, West Lafayette, IN, 47907 USA

[b]Elmore Family School of Electrical and Computer Engineering, Purdue University, West Lafayette, IN 47907, USA

*Corresponding author: jdick@purdue.edu

Table of Contents **Pages**

1. Experimental Section **S2**
2. Example of Chronoamperogram Recorded During an ECL Imaging Experiment **(Figure S1)** **S3**
3. Example of ECL Images Sequences Obtained with a Pt as working electrode material (**Figure S2**) **S4**
4. Bright-field Images of the Water droplets in Figure 2, Before and After Coalescence **(Figure S3)** **S5**
5. Example of ECL Images Sequences **(Figure S4, Figure S5)** **S6-S7**
6. Cobalt Deposition Experiment and SEM Images **(Figure S6)** **S8**

References **S9**

1.Experimental Section

Chemicals. 1,2-Dichloroethane (99.8%), tetrabutylammonium perchlorate (99%), sodium oxalate (99%), and cobalt (II) chloride hexahydrate (98%) were purchased from Sigma Aldrich. Tris(bipyridine)ruthenium (II) chloride hexahydrate ($[Ru(bpy)_3][Cl_2] \cdot 6\ H_2O$, 98%) was purchased from Acros Organics. Potassium chloride was purchased from Fisher Bioreagents. All chemicals were used as received. Aqueous solutions were prepared from ultrapure H_2O (>18 MΩ·cm, Thermo Scientific).

Water-in-DCE emulsion preparation procedure. The emulsion was prepared following a previously published method.[1] Briefly, an aliquot of 100 µL of aqueous solution containing 50 mM of sodium oxalate and 10 mM of Tris(bipyridine)ruthenium (II) chloride hexahydrate was added to 5 mL of a DCE/$[TBA][ClO_4]$ 0.1 M in a 20 mL glass scintillation vial. For the cobalt deposition experiment, 50 µL of aqueous solution containing 10 mM of cobalt (II) chloride hexahydrate and 1 M of potassium chloride was added to 5 mL of a DCE/$[TBA][ClO_4]$ 0.1 M in a 20 mL glass scintillation vial. The two-phases solution was then ultrasonicated (500 W, 40% amplitude) with a pulsing method (5 s on 5 s off, 10 cycles).

Optical and ECL microscopy. The imaging system was composed by a Leica Dmi8 microscope (Leica Microsystems, Germany) equipped with a Lambda LS xenon arc lamp (Sutter Instrument Company, Novato, CA), a Leica CTR advanced electronics box (Leica Microsystems, Germany), a Leica SP box LMT200 (Leica Microsystems, Germany), a Complementary Metal-Oxide Semiconductor camera (Orca-Quest Qc-MOS C15550-20UP from Hamamatsu, Hamamatsu Japan), a Lambda SC SmartShutter controller (Sutter Instrument Company, Novato, CA), two Lambda VF-5 tunable filter changers (Sutter Instrument Company, Novato, CA) and a Lambda 10-3 optical filter changer and SmartShutter control system (Sutter Instrument Company, Novato, CA). The objectives used during the imaging experiments were a 5x, NA 0.12, 1.4 cm free working distance for Figures 2 and 3 and 63x, NA 1.4, working distance 0.14 mm for Figures S3 and S4. ECL reactants were electrogenerated by performing chronoamperometry at a 601E Electrochemical Workstation (CH Instruments, Austin, TX) with a Ag/AgCl (saturated KCl) as reference electrode (RE). The RE had a Vycor frit sealed with a Teflon wrap for compatibility with DCE. The glassy carbon working electrode (r = 1.5 mm) was polished on a 10,000-grit polishing pad (Buehler, Lake Bluff, IL) with 0.05 µm alumina powder, followed by polishing on a clean polishing cloth to remove residual alumina powder. After polishing, the electrode was rinsed with water, ethanol and then DCE. A glassy carbon rod was used as the counter electrode.

SEM and EDX Data. A Helios 600 Nanolab dual beam system (FEI, Hillsboro, OR) was used to collect scanning electron microscopy (SEM) images. An INCA PentaFet-x3 detector (Oxford Instruments, Abingdon, UK) was used to perform energy dispersive X-ray spectroscopy (EDX). All SEM images and EDX data were collected with 20 keV and 0.69 nA beam.

2. Example of Chronoamperogram Recorded During an ECL Imaging Experiment

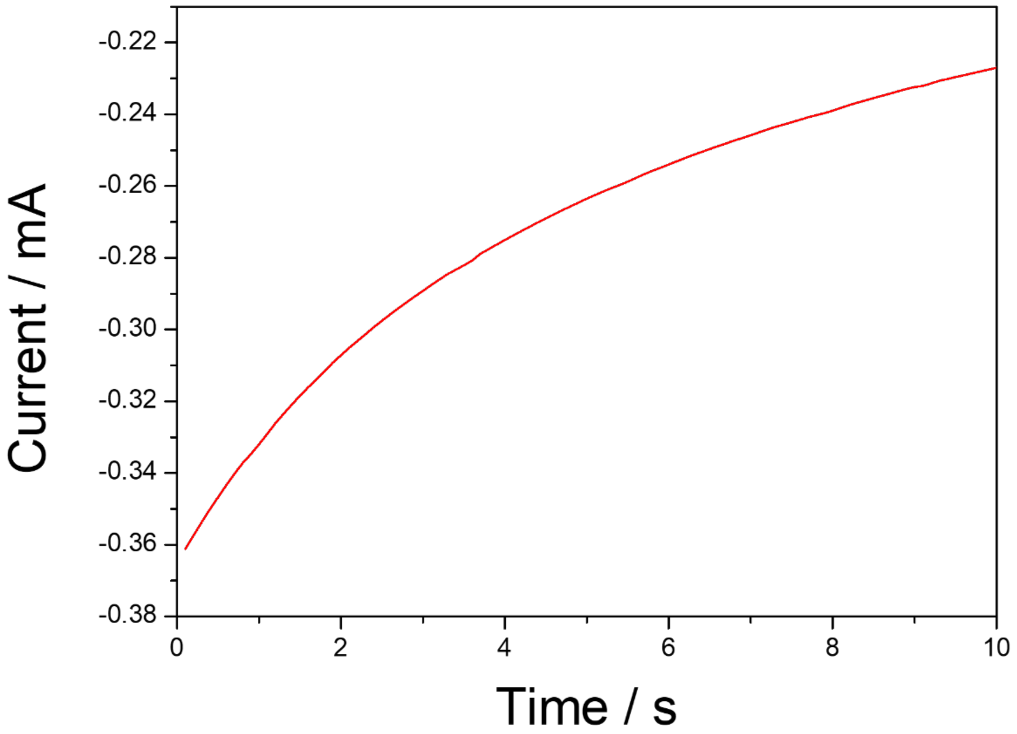

Figure S1 Chronoamperogram recorded by applying 1.5 V *vs* Ag/AgCl on a glassy carbon working electrode (d = 3 mm) in an emulsion composed of an aqueous solution of 10 mM tris(bipyridine)ruthenium(II) dichloride and 50 mM sodium oxalate suspended in a continuous phase of 1,2-dichloroethane/0.1 M tetrabutylammonium perchlorate.

3. Example of ECL Images Sequences Obtained with a Pt as working electrode material

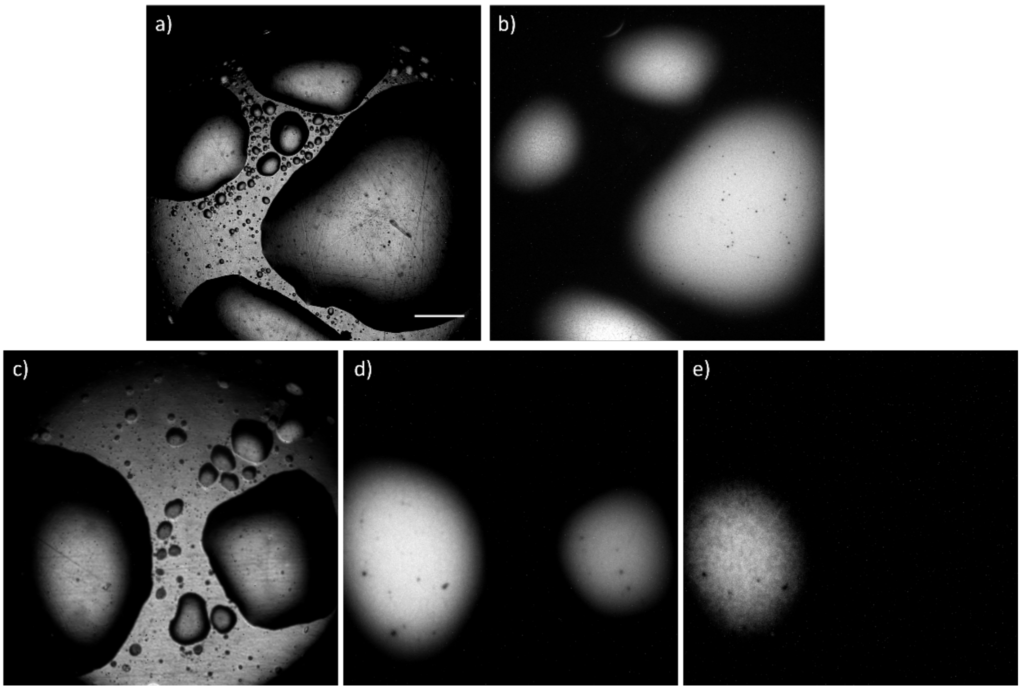

Figure S2 a) Optical and b) ECL image of water droplets loaded with 10 mM tris(bipyridine)ruthenium(II) dichloride and 50 mM sodium oxalate in 1,2-dichloroethane with 0.1 M tetrabutylammonium perchlorate continous phase; c) optical and d) and e) ECL images in sequence water droplets loaded with 10 mM tris(bipyridine)ruthenium(II) dichloride and 50 mM sodium oxalate in 1,2-dichloroethane with 0.1 M tetrabutylammonium perchlorate continous phase. ECL images were obtained by applying 1.5 V vs Ag/AgCl on a platinum working electrode for 10 seconds of exposure time. Scale bar: 500 µm.

4. Bright-field Images of the Water droplets in Figure 2, Before and After Coalescence

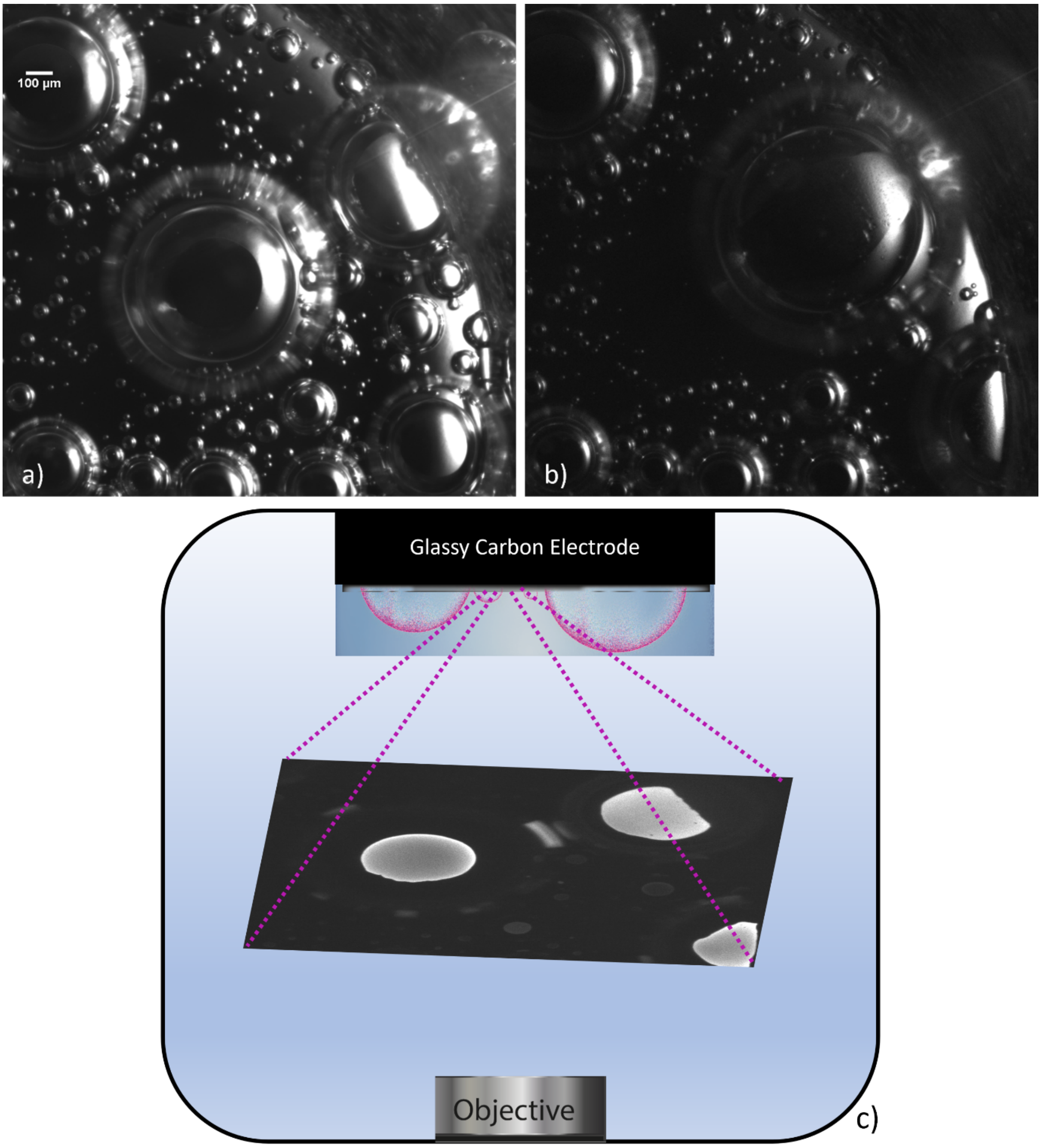

Figure S3 Bright-field images recorded before a) and after b) the coalescence event reported as ECL images in **Figure 2**; c) schematic representation of the ECL imaging experiment reported in **Figure 2.**

5. Example of ECL Images Sequences

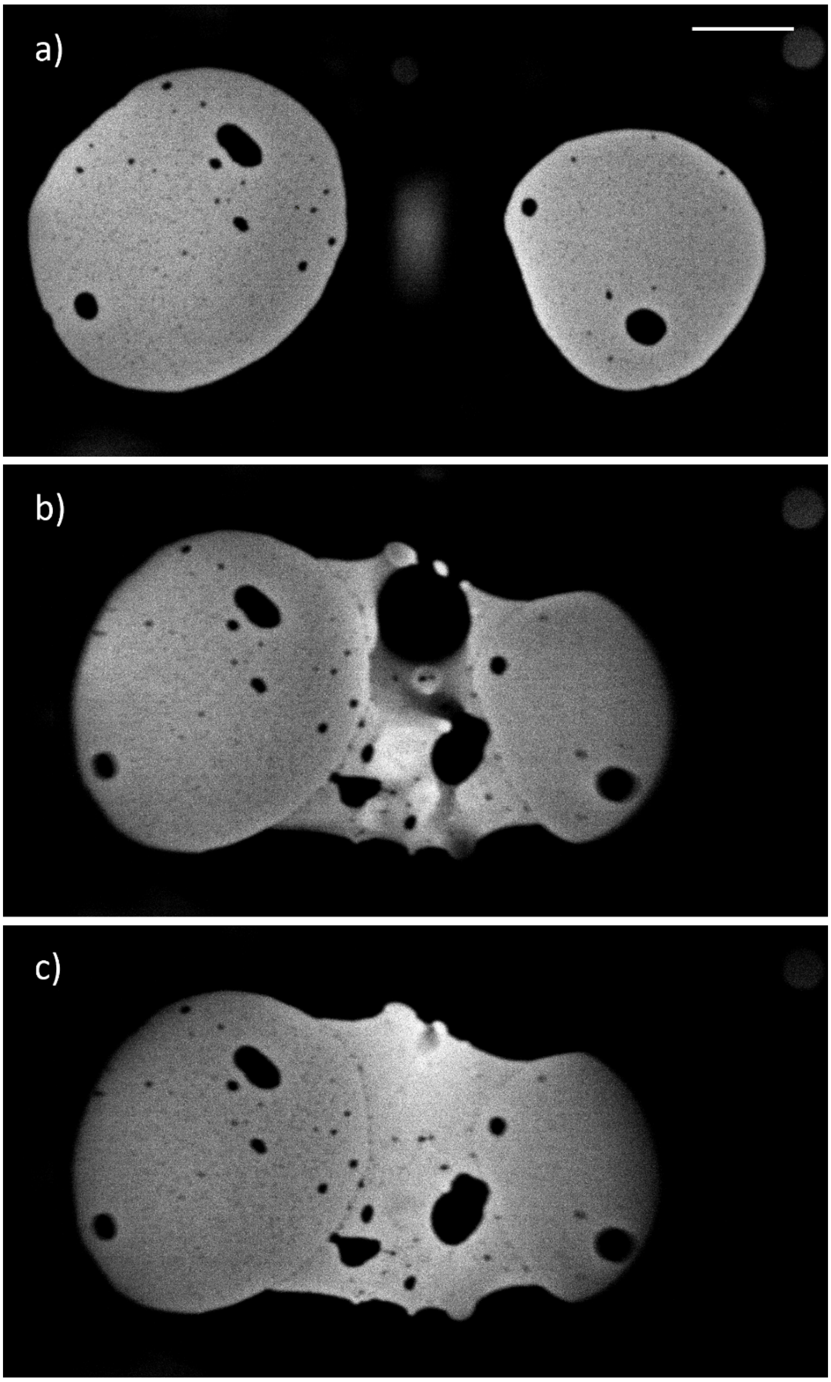

Figure S4 ECL images in sequence of two water droplets loaded with 10 mM tris(bipyridine)ruthenium(II) dichloride and 50 mM sodium oxalate in 1,2-dichloroethane with 0.1 M tetrabutylammonium perchlorate continous phase. ECL images were obtained by applying 1.5 V vs Ag/AgCl on the glassy carbon working electrode for 500 milliseconds of exposure time. Scale bar: 20 μm.

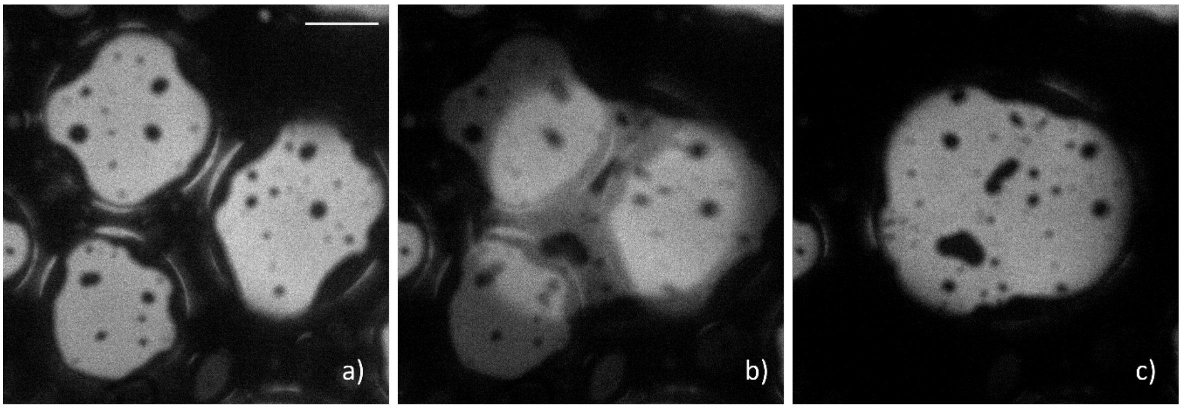

Figure S5 ECL images in sequence of three water droplets loaded with 10 mM tris(bipyridine)ruthenium(II) dichloride and 50 mM sodium oxalate in 1,2-dichloroethane with 0.1 M tetrabutylammonium perchlorate continous phase. ECL images were obtained by applying 1.5 V vs Ag/AgCl on the glassy carbon working electrode for 500 milliseconds of exposure time. Scale bar: 20 μm.

6. Cobalt Deposition Experiment and SEM Images

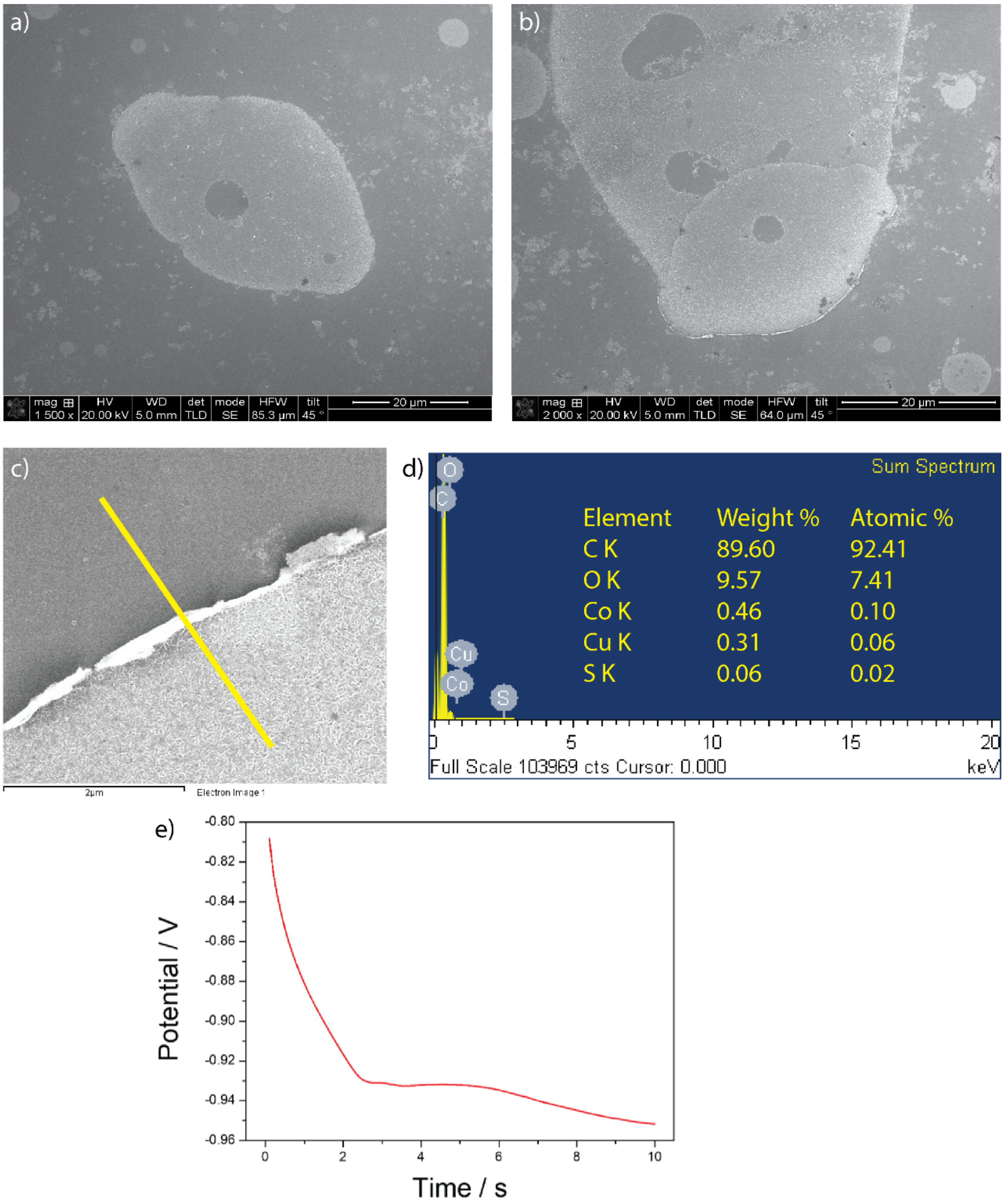

Figure S6 Cobalt deposition experiment where 50 µL of 10 mM $CoCl_2•6H_2O$ + 1M KCl was suspended in a 1,2-dichloroethane solution of 0.1 M tetrabutylammonium perchlorate. For deposition, 50 µA of cathodic current was applied for 10 s across the glassy carbon (d = 3 mm) working electrode using chronopotentiometry. Scanning electron microscopy images (a), b), and c)) showing regions of cobalt deposition with clear inclusions preventing deposition. Energy dispersive X-ray spectrum (d)) collected from the line segment in c), showing the elemental composition of the deposited material. Chronopotentiogram (e)) measured during the passage of 50 µA of cathodic current for 10 s using a glassy carbon (d = 3 mm) working electrode, with potentials measured *vs* a Ag/AgCl reference electrode.

References

1. Glasscott M., Pendergast A.D., Goines S., Bishop A.R., Hoang A.T., Renault C., Dick J.E., *Nat. Comm.*, **2019**, *10*, 2650.
